# Factors associated with non-pathogenic antibodies against desmoglein-3 in pemphigus foliaceus^☆^

**DOI:** 10.1016/j.abd.2023.11.006

**Published:** 2024-06-08

**Authors:** Sebastian Vernal, Tamiris Amanda Julio, Fernando Henrique Alves, Aline Turatti, Eduardo Antonio Donadi, Ana Maria Roselino

**Affiliations:** a Laboratory of the Dermatology Division, Hospital das Clínicas, Faculty of Medicine of Ribeirão Preto, Universidade de São Paulo, São Paulo, SP, Brazil; b Division of Dermatology, Department of Internal Medicine, Faculty of Medicine of Ribeirão Preto, Universidade de São Paulo, São Paulo, SP, Brazil; c Division of Immunology, Department of Internal Medicine, Faculty of Medicine of Ribeirão Preto, Universidade de São Paulo, São Paulo, SP, Brazil

**Keywords:** Desmoglein 1, Desmoglein 2, Desmoglein 3, Endemic Pemphigus Foliaceus, HLA-DRB1 Chains, Pemphigus

## Abstract

**Background:**

Anti-desmoglein (Dsg)1 is produced in pemphigus foliaceus (PF), affecting exclusively the skin. Pemphigus vulgaris (PV) shows the production of anti-Dsg3 in the mucosal form, and anti-Dsg1 and 3 in the mucocutaneous form. Anti-Dsg3 autoantibodies have been rarely reported in PF.

**Objectives:**

To determine the factors associated with the production and pathogenicity of anti-Dsg3 in PF.

**Methods:**

Comparative analytical study of three patients groups: 16 PF-anti-Dsg3+, and 42 PF-anti-Dsg3(-) and 22 PV treatment-naïve cases. Serum was used in the anti-Dsg1 and 3 ELISA, and in immunoblotting (IB) with human epidermis extract. The expression of Dsg1 and 3 in paraffin sections was analyzed by immunohistochemistry (IHC). *HLA-DRB1* alleles were compiled from a database.

**Results:**

In the PF-anti-Dsg3+ group: age range similar to that of the PV group (p > 0.9999); predominance of the generalized form of PF (p = 0.002); anti-Dsg3 titers lower than those of PV (p < 0.0001); IB confirmed Dsg3 identification in one (8.33%) of 12 patients; IHC showed exclusive cytoplasmic internalization of Dsg1; *HLA-DRB1* alleles of susceptibility to PF, with the absence of alleles associated with PV, in the five typed patients.

**Study limitations:**

Most of the patients in the PF-anti-Dsg3+ group were undergoing treatment.

**Conclusion:**

The presence of anti-Dsg3 antibodies in PF was related to older age (comparable to that of PV) and the generalized form of PF. The non-pathogenicity of anti-Dsg3 antibodies in PF can be attributed to the low serum anti-Dsg3 titers, the lack of Dsg3 internalization as detected by IHC, and the absence of PV-associated *HLA-DRB1* alleles.

## Introduction

Pemphigus are autoimmune diseases, in which intraepidermal acantholytic bullae are caused by the deposition of serum antibodies in desmogleins (Dsg), components of the epidermal desmosomes.^1^, ^2^, ^3^ Pemphigus vulgaris (PV) and pemphigus foliaceus (PF) are prevalent in southeastern Brazil.^4^, ^5^ In PV, patients are usually elderly, with initial mucosal involvement, and have autoantibodies against Dsg3. In the mucocutaneous form of PV, there is also the production of anti-Dsg1. PF (also known as *Fogo Selvagem* [FS] in Brazil) affects exclusively the skin of young adults, with anti-Dsg1 production.^1^, ^2^, ^3^, ^4^, ^5^

Dsg1 and 3 are transmembrane proteins of 160kDa and 130kDa, respectively, expressed by genes located on chromosome 18.^6^ Dsgs compensation theory explains the level of intraepidermal acantholysis in pemphigus.^7^ Anti-Dsg1 antibodies in PF cause subgranular acantholysis, as Dsg1 is expressed more intensily in the upper layers of the epidermis. Anti-Dsg3 antibodies cause suprabasal acantholysis, as Dsg3 is most often expressed in the lower layers of the epidermis, and in all epithelial layers in the mucous membranes. Moreover, as there is a lower expression of Dsg1 in the mucosa, it is not affected by anti-Dsg1 antibodies in PF, as Dsg3 compensates for epithelial cell cohesion in the mucosa.^8^ Subsequently, the hypothesis of Dsgs compensation, to explain the level of cleavage of epidermal acantholysis related to the production of anti-Dsg1 and 3, started to be discussed again, since exclusively suprabasal acantholysis in PV would not be explained in the mucocutaneous form, when anti-Dsg1 is also present. Other target molecules of epidermal adhesion are now identified in the pathogenesis of pemphigus, as well as mitochondrial proteins, cholinergic receptors, and other molecules, which act synergistically with anti-Dsg1 and Dsg3 antibodies in epidermal acantholysis.^9^, ^10^, ^11^, ^12^, ^13^, ^14^, ^15^, ^16^, ^17^, ^18^

As both PV and PF are prevalent in southeastern Brazil, there is an opportunity for outpatient follow-up of a large series of patients.^4^, ^5^ In the PF series, over a 25-year period, it was observed that 6,64 % of the patients, in addition to being positive for anti-Dsg1, also had reactive titers to Dsg3 in the ELISA test, without, however, showing a phenotype of mucosal lesions. Therefore, we aimed to analyze clinical-laboratory data in the group of patients with PF and reactivity to Dsg3 (PF-anti-Dsg3+). For this purpose, three groups were compared: PF-anti-Dsg3+, PF with anti-Dsg3 negativity and treatment-naïve [PF-anti-Dsg3(˗)], and treatment-naïve PV. For specific purposes, the following were evaluated: (i) Demographic and clinical data; (ii) Temporal evolution of anti-Dsg1 and 3 titers by ELISA and identification of Dsg1 and 3 by immunoblotting (IB) in the PF-anti-Dsg3+ group; and (iii) Expression of Dsg1 and 3 in paraffin-embedded skin biopsies by immunohistochemistry (IHC) and (iv) typification of *HLA-DRB1* alleles associated with PF and PV in the PF-anti-Dsg3+ and PF-anti-Dsg3(-) groups.

## Methods

This is a comparative analytical study of three groups. The sample bank of the Dermatology Laboratory, Hospital das Clínicas, FMRP-USP, has the approval of the Research Ethics Committee (HCRP Process number 3605/2006). Patients and control individuals signed the free and informed consent form at the time of sample collection.

### Case series

It consisted of 80 patients: 16 PF-anti-Dsg3+, 42 PF-anti-Dsg3(-) and 22 PV cases. The clinical findings were confirmed by histopathological examination and by direct immunofluorescence (DIF) and/or indirect immunofluorescence (IIF). Demographic, clinical and laboratory data (anti-Dsg1 and Dsg3 by ELISA and HLA alleles) were obtained from data recorded at the Laboratory of Dermatology. In the PF-anti-Dsg3+ group, three (20%) of the 15 patients (with information about treatment) were untreated at the time of blood collection, while 42 of the PF-anti-Dsg3(-) patients and 22 PV were all treatment-naïve. Immunoblotting (IB) assays were performed with 12 (75%) of the 16 serum samples from the PF-anti-Dsg3+ group, with some patients having more than one sample analyzed, collected at different times. IHC was performed on sections from the paraffin blocks of four patients from the PF-anti-Dsg3+ group and one from the PF-anti-Dsg3(-) group.

### ELISA for the detection of IgG antibodies against Dsg1 and 3

The manufacturers recommendations were followed. Values ≥ 20 U/mL, between 9‒20 U/mL and < 9 U/mL were considered positive, indeterminate and negative, respectively (MBL, Nagoya, Japan).

### IB with human epidermis extract and patient serum

Briefly, skin fragments were incubated for 48 h in PBS with EDTA (Merck) and PMSF (Sigma-Aldrich) to separate the epidermis from the dermis. Protein extraction from the epidermis was carried out with a Tris-HCL, SDS, 2-mercaptoethanol solution, and then an EDTA, PMSF and proteinase inhibitor cocktail (Sigma-Aldrich) were added. After the electrophoretic run, the polyacrylamide gel was assembled in a sandwich with a 0.45 µm nitrocellulose membrane (BioRad) for protein transfer. After blocking with 3% skimmed milk in TBS, and consecutive washings, the serum samples (1:20) were incubated on nitrocellulose strips, followed by incubation with anti-human HRP IgG secondary antibody (BioRad). After washing, color reagent (Color-Plus HRP, BioRad) was added for colorimetric development.

### Expression of Dsgs1 and 3 in paraffin-embedded samples with IHC

For IHC assays, the manufacturers recommendations were followed (HRP-Polymer MACH1, Biocare Medical, Concord, CA, USA). Briefly, 5μm sections were obtained and after antigen retrieval in citrate, inhibition of endogenous peroxidase and use of a blocking solution, were incubated with anti-Dsg1 and 3 monoclonal antibodies produced in mice (Abcam, MA, USA). After washing and incubation with Probe and HRP polymer, the reaction was terminated with diaminobenzidine (DAB).

### Statistical analysis

The GraphPad Prism 9.2.0 software was used for statistical analysis and the generation of graphs. Frequencies between the groups were analyzed using Fisher or Chi-Square tests, and numerical data were analyzed using Kruskal-Wallis tests followed by Dunn’s multiple comparisons. The result was considered significant when p≤0.05.

## Results

### Demographic and clinical data (Table 1)

There was no statistical difference regarding gender in the three groups. As for age groups, the PF-anti-Dsg3(-) group was younger than the PV group (p = 0.0522). There was no statistical difference when comparing the PF-anti-Dsg3+ with the PF-anti-Dsg3(-) and the PV group (p = 0.5361 and p > 0.9999, respectively). The generalized form predominated in the PF-anti-Dsg3+ group, when compared to the PF-anti-Dsg3(-) group (p = 0.002).

**Table 1:** Demographic, clinical and antibody against Dsg1 and 3 data of the PF-anti-Dsg3+ group, compared to the PF-anti-Dsg3(-) and PV groups.

		PF-anti-Dsg3+	PF-anti-Dsg3(-)	PV	p
		n = 16	n = 42	n = 22	
**Gender**	Male	11	26	10	0.296
	Female	5	16	12	
**Age (years)**	Median (minimum‒maximum)	40.5 (12‒74)^a^	32 (12‒71)^b^	50 (13‒72)^c^	^a^·^b^0.5361; ^a^·^c^>0.9999; ^b^·^c^0.0522
**Clinical form**	Localized	3	24	NA	0.002
	Generalized	13	11		
	U	0	7		
**Treatment**	Treatment-naïve	2	42	22	NA
	No treatment >60 days	1			
	Undergoing treatment	12			
	U	1			
**Anti-Dsg1 (UR/L)^#^**	Median (25%‒75% quartiles)	189.0 (135.1‒254.5)^a^	166.9 (121.7‒203.7)^b^	40.94 (3.3‒99.4)^c^	^a^·^b^0.4515; ^a^·^c^<0.0001; ^b^·^c^<0.0001
**Anti-Dsg3 (UR/L)^#^**	Median (25%‒75% quartiles)	33.1 (23.2‒63.0)^a^	0.688 (0.3‒1.6)^b^	128.6 (56.6‒168.6)^c^	^a^·^b^0.0002; ^a^·^c^<0.0001; ^b^·^c^<0.0001

^#^Series: n = 16, n = 39 and n = 21, respectively; cut-off level for positivity ≥ 20 U/mL.

U, Unavailable; NA, Not Applicable.

### Serological data

**Figure 1:**
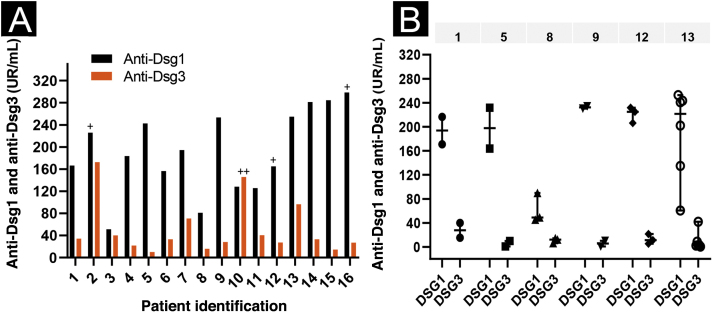
**Anti-Dsg1 and anti-Dsg3 titration by ELISA in the PF-anti-Dsg3+ group.** Values ≥ 20 U/mL, between 9‒20 U/mL and < 9 U/mL were considered positive, indeterminate and negative, respectively (MBL, Jp). (A) Serum titration of the 16 patients in the first appointment. Patients PF5 and PF14: serum was collected while treatment-naïve; PF6, without treatment for more than 60 days; PF3, treatment not included; the others were undergoing treatment. Patients PF4, PF5 and PF7: localized clinical form, the others, generalized form. The + and ++ symbols above the bars indicate serum positivity in the IB (Fig. 2). (B) Serial titration of six patients. The line above the graph shows the number of patients. Each symbol corresponds to a serum sample collected on consecutive dates; the vertical lines show the variation in titles (minimum and maximum); and the horizontal line, the median. Observe the slight variation in anti-Dsg3 titers.

Regarding anti-Dsg3 antibodies, the PF-anti-Dsg3+ group had lower titers than the PV group (p < 0.0001; Table 1). The distribution of anti-Dsg3 titers in the PF-anti-Dsg3+ group is depicted in Fig. 1A. Fig. 1B shows slight variation over time in anti-Dsg3 titers in six patients.

**Figure 2:**
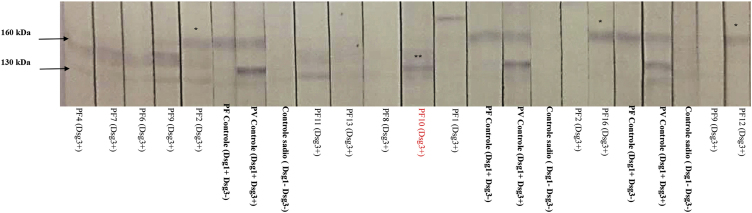
**Immunoblotting with serum samples from 12 patients in the PF-anti-Dsg3+ group (some with consecutive samples) and protein extract from human epidermis.** Positive controls correspond to samples of pemphigus foliaceus (PF), which recognize Dsg1 (160 kDa), and pemphigus vulgaris (PV), which recognizes Dsg1 and 3 (130 kDa). Negative controls correspond to samples from healthy individuals. Observe the nitrocellulose strips with the 160 kDa band (*) corresponding to patients PF2, PF12 and PF16, and the 130 kDa band (**) corresponding to PF10. For ELISA titers of anti-Dsg1 and anti-Dsg 3 seeFig. 1A.

In the IB performed with sera from 12 patients, three recognized Dsg1, and one recognized Dsg3 (Fig. 2). PF10, which recognized Dsg3 in the IB, showed the 2nd highest anti-Dsg3 titer in the ELISA (Fig. 1A).

### Expression of Dsgs 1 and 3 with IHC (Fig. 3)

**Figure 3:**
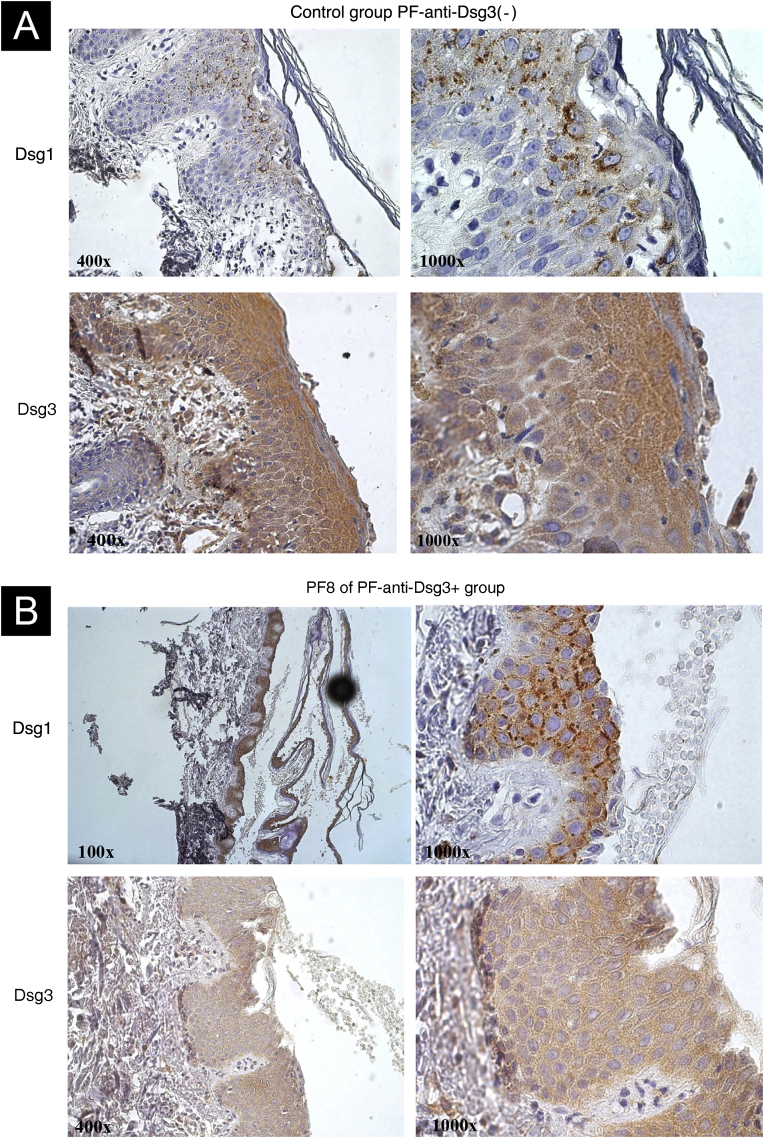
**IHC images with Dsg1 and Dsg3 expression stained with DAB in paraffin sections from bullous lesion biopsies showing superficial acantholysis.** (A) Control sample from the PF-anti-Dsg3(-) group. Observe Dsg1 expression in intracytoplasmic clumps, and Dsg3 expression throughout the epidermis. (B) Sample from patient PF8 in the anti-Dsg3+ group. The Dsg1 and Dsg3 expressions are similar to those of the PF control.

IHC showed internalization of Dsg1, with coarse clumps inside the cytoplasm, sometimes perinuclear, in skin samples from patients in the PF-anti-Dsg3(-) (Fig. 3A) and PF-anti-Dsg3+ (Fig. 3B). Dsg3 expression occurred in the keratinocyte envelope and intracytoplasmically, with emphasis on its expression throughout the epidermis. No internalization of Dsg3 was observed.

### *HLA-DRB1* alleles associated with PF and PV in the PF-anti-Dsg3+ and PF-anti-Dsg3 (-) groups; Table 2)

Five (31.3%) of 16 patients in the PF-anti-Dsg3+ group, and 16 (38.1%) of 42 in the PF-anti-Dsg3(-) group were typed. In the PF-anti-Dsg3+ group, five patients showed one or two susceptibility alleles for PF, and none of them had alleles associated with PV. In the PF-anti-Dsg3(-) group, 11 had alleles of susceptibility to PF, and two patients had alleles of susceptibility to PV in heterozygous form.

**Table 2:** Distribution of *HLA-DRB1* alleles associated with pemphigus foliaceus (PF) and pemphigus vulgaris (PV) in the groups PF-anti-Dsg3+ and PF-antiDsg3(-).

^#^Patients with higher anti-Dsg3 titers (Fig. 1A). In orange and yellow: susceptibility alleles to PF in the northeastern region of the state of São Paulo and in other regions of Brazil, respectively. In dark green: PF resistance allele described in other regions of Brazil. In red and light green: susceptibility and resistance alleles to PV, respectively, in the northeastern region of the state of São Paulo. PF and PV association alleles were collected from Franco Brochado et al., 2016^36^ and Franco Brochado et al., 2016.^37^

## Discussion

The production of serum autoantibodies against Dsg3 is not expected in PF, since, in its pathogenesis, only anti-Dsg1 antibodies are implicated in subgranular acantholysis, with consequent formation of flaccid bullae on the skin. The PF phenotype does not include the mucosal lesions observed in PV due to antibodies against Dsg3.^1^, ^2^, ^3^, ^4^, ^5^ However, autoantibodies against Dsg3 have been rarely reported in PF cases.^19^, ^20^, ^21^ More recently, anti-Dsg3 antibodies have been reported in other forms of pemphigus.^22^, ^23^ Arteaga et al. (2002) described anti-Dsg3 in 7% of 276 patients with PF, demonstrating that anti-Dsg3 antibodies did not show a serological cross-reaction with Dsg1.^19^ Flores et al. (2012) reported anti-Dsg3 in 40% of 101 FS sera, and in 14% of controls from an endemic region for FS in Brazil.^20^ Oliveira et al. (2016) reported anti-Dsg3 in 4% of the patients in a PF series (including patients from the northeastern region of the state of São Paulo).^21^ The present series confirmed 6.64% of patients with PF with indeterminate or positive results for anti-Dsg3 in the ELISA test.

When comparing the three groups, the age range of the PF-anti-Dsg3+ group tended to be older, comparable to that of the PV group (p>0.999), and similar to that of the PF-anti-Dsg3(-) group. The generalized clinical form predominated in the PF-anti-Dsg3+ group (p=0.0020). Regarding the anti-Dsg3 titers of the PF-anti-Dsg3+ group, they were lower when compared to those of the PV group (p<0.0001). The measurement of anti-Dsg3 in the PV group was carried out in treatment-naïve patients, while in the PF-anti-Dsg3+ group, three (20%) of the 15 patients (with information on treatment) were not receiving treatment (PF5, PF6 and PF14). Anti-Dsg3 titers in the PF-anti-Dsg3+ group showed no difference between treated and untreated patients (Fig. 1A). Two patients stand out, in the sample of 16, with higher anti-Dsg3 titers, whose blood was collected while undergoing treatment. Patient PF2, a 63-year-old male, presented the generalized form, with 14 years evolution, and had keratoacanthoma and melanoma during the follow-up. Another patient PF10, 46 years-old male, had the generalized form, and a two-year history. There were coinciding factors in both cases, in addition to higher anti-Dsg3 titers, male gender, having had the onset of PF at an older age, and the generalized form of the disease. IB confirmed the production of anti-Dsg3 only in PF10 (Fig. 2).

The internalization of Dsgs, in the process of acantholysis, is observed on IHC by the formation of intracytoplasmic and perinuclear granules in keratinocytes, when anti-Dsg1 and 3 antibodies are used in PF and PV, respectively.^24^ The internalization of Dsg1 was observed in samples from the PF-anti-Dsg3(-) and PF-anti-Dsg3+ groups; however, there was no internalization of Dsg3. The low anti-Dsg3 titers in the PF-anti-Dsg3+ group could justify the lack of Dsg3 internalization, as well as the absence of mucosal lesions. In exclusively cutaneous PV, low anti-Dsg3 titers could explain the absence of mucosal lesions.^25^

Although *HLA-DRB1* alleles were not typed in the whole group of PF-anti-Dsg3+, exclusive alleles of susceptibility to PF were determined in the five individuals that were typed, in homozygous or heterozygous forms, without the presence of alleles associated with PV. Recently, Sielski et al. (2022) demonstrated that cases of PV that contradicted the hypothesis of Dsgs compensation were related to the absence of *DRB1* alleles of susceptibility to PV.^26^ Therefore, the absence of susceptibility alleles to PV in the PF-anti-Dsg3+ group could contribute to the non-pathogenicity of antibodies against Dsg3.

The production of anti-Dsg3 in PF could be justified by the phenomenon of epitope spreading ‒ patients with a specific bullous disease have non-pathogenic autoantibodies against other molecules of the epidermis that do not cause the specific bullous disease. Its pathogenesis is explained by the exposure of other epidermal molecules during the inflammation process caused by acantholysis, in the case of pemphigus.^27^, ^28^, ^29^, ^30^ In this study, IHC showed the expression of Dsg3 in all layers of the epidermis. Thus, it could justify the greater exposure of Dsg3, with consequent production of anti-Dsg3. However, this expression in all layers of the epidermis was observed in samples from both groups – PF-anti-Dsg3(-) and PF-anti-Dsg3+. As the PF-anti-Dsg3(-) group consisted of treatment-naïve patients, it is not known whether anti-Dsg3 would be produced during pemphigus evolution.

Moreover, there are rare reports of patients with clinically and laboratory-proven PF who migrate to the PV phenotype, or of patients with both PF and PV characteristics.^31^, ^32^, ^33^, ^34^ However, PV migrating to PF is more commonly observed.^35^ The present case series does not show similar cases.

## Conclusions

The presence of anti-Dsg3 antibodies in PF was related to an older age group (comparable to that of PV) and the generalized form of PF. The non-pathogenicity of anti-Dsg3 antibodies in PF can be attributed to low anti-Dsg3 titers, the lack of Dsg3 internalization as seen on IHC, and the absence of PV-associated *HLA-DRB1* alleles.

## Financial support

The project was funded by 10.13039/501100001807FAPESP (Fundação de Amparo à Pesquisa do Estado de São Paulo) (#2010/51729-2), SV received a PhD scholarship from 10.13039/501100002322CAPES (Coordination for the Improvement of Higher Education Personnel) and TAJ received a PhD scholarship from FAPESP.

## Authors’ contributions

Sebastian Vernal: Contributed to data survey, collection and interpretation of data; responsible for blotting assays.

Tamiris Amanda Julio: Contributed with data survey, collection and interpretation of data; responsible for the ELISA assays.

Fernando Henrique Alves: Contributed with data survey and collection of data, and participation in the propaedeutic and therapeutic conduct, and in IB assays.

Aline Turatti: Contributed with data survey, collection and interpretation of data; responsible for DIF, IIF and IHC assays.

Eduardo Antonio Donadi: Contributed with data survey, collection and interpretation of data; responsible for determining *HLA-DR* alleles.

Ana Maria Roselino: Contributed with the design and planning of the study, collection and analysis of data; drafting and editing of the manuscript; participation in the propaedeutic and/or therapeutic conduct; and critical review of the literature.

All authors approved the final version of the manuscript.

## Conflicts of interest

None declared.
